# Magnetically recoverable cobalt oxide nanoparticle catalyst for PET glycolysis

**DOI:** 10.1039/d5ra05618g

**Published:** 2025-11-05

**Authors:** Deepthi Thomas, Parvathy C., Benny K. George

**Affiliations:** a Vikram Sarabhai Space Centre, Indian Space Research Organization Thiruvananthapuram 695022 Kerala India bkgeorge63@gmail.com; b Department of Applied Chemistry, Cochin University of Science and Technology Cochin 682022 India

## Abstract

In spite of its many applications and desirable properties, the non-biodegradable nature of polyethylene terephthalate (PET) raises concerns about the accumulation of post-consumer products. Recycling these end-of-life polymers is the most effective solution to address this issue. Mechanical recycling, the primary commercial recycling method for PET, often produces materials with inferior properties, and hence chemical recycling, or tertiary recycling, is considered a viable alternative. Glycolysis, which is a transesterification reaction in which the PET molecule is depolymerized to its monomer, bis(hydroxyethyl) terephthalate (BHET), has gained significant commercial interest due to its mild reaction conditions. In the present work, magnetic cobalt oxide nanoparticles (CONP) were synthesized using different methods: NaBH_4_ reduction route, hydrothermal route, and combustion route, and experimented as catalysts for the glycolysis of PET. The catalysts produced were characterized using FTIR, SEM-EDS, surface area and XRD techniques. Glycolysis of PET was performed with the CONP catalysts obtained from each synthesis method and the resulting BHET was analysed using DSC, HPLC, and NMR techniques. Among the catalysts, CONP prepared *via* the NaBH_4_ route showed the best performance, achieving a BHET yield of 97% with just 1% catalyst at 180 °C within a reaction time of 2 hours. The effects of various reaction conditions, including temperature, reaction time, and the PET/EG ratio, were also investigated. Notably, the CONP–NaBH_4_ catalyst is magnetically separable from the reaction mixture after the process and can be reused multiple times without considerable loss in efficiency. CONP–NaBH_4_ is structurally distinct from other cobalt oxides, featuring a core–shell structure with a nanocrystalline cobalt boride core and an amorphous cobalt oxide, oxyhydroxide, hydroxide shell. This was established by STEM, HRTEM, XPS, Raman, and FTIR analyses. The amorphous cobalt oxide shell drives catalytic activity, while the cobalt boride core imparts ferromagnetism. Superior catalytic performance is attributed to its high surface area, porosity, and presence of surface hydroxyl groups.

## Abstract 2

A magnetically recoverable Co–B/CONP core–shell catalyst with 97% BHET yield, reported for the first time. The unique core–shell structure contributes to the inherent ferromagnetism, while its high surface area enhances catalytic performance.

## Graphical abstract

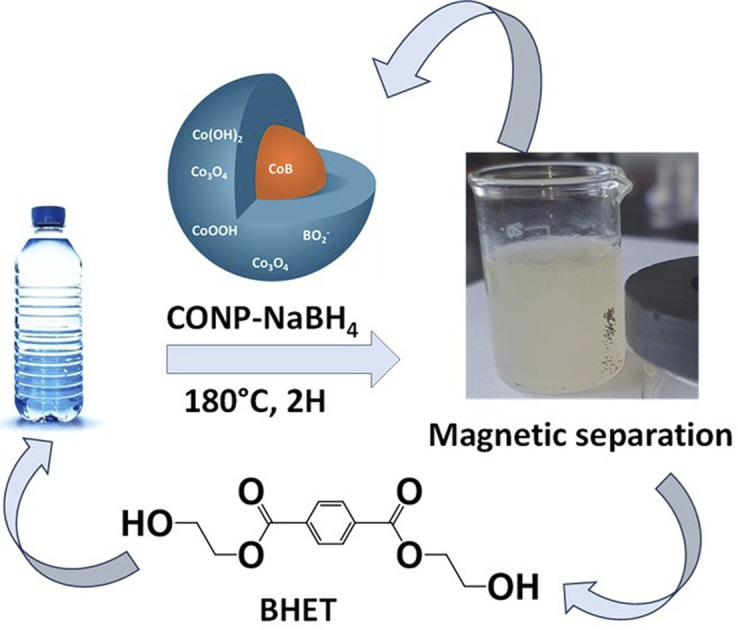

## 1. Introduction

Polymers and polymer-based products are an integral part of our daily lives, with applications ranging from household appliances to aerospace materials. In recent decades, use of polymer products has surged globally due to their diverse applications.

Polyethylene terephthalate (PET), a polyester made from terephthalic acid and ethylene glycol is utilized in various fields, including food packaging and aerospace. However, PET's non-biodegradable nature raises concerns about the accumulation of post-consumer PET products in the environment. Recycling end-of-life polymers is a viable solution to address this issue. Recycling strategies reported for PET waste are re-extrusion (primary recycling), mechanical recycling (2° recycling), and tertiary or feedstock recycling. Though mechanical recycling is the major route of recycling, it always results in a downgrade product.^1,2^ Among the various methods for tertiary recycling of PET, glycolysis has gained commercial interest due to its favourable reaction conditions. Glycolysis is a transesterification reaction that depolymerizes the PET molecule using ethylene glycol to produce bis(hydroxyethyl) terephthalate (BHET). BHET can be used as a starting material for PET polymerisation or to produce other value-added products like polyurethane or acrylates.^1^

PET glycolysis is a slow process without a catalyst. The catalysts used for PET glycolysis fall into several categories as: metal derivatives,^3–6^ ionic liquids (IL),^7–10^ deep eutectic solvents (DES),^11–13^ and organic catalysts.^14,15^ Metal-based catalysts include metal salts,^16,17^ metal oxides,^6,18^ metal–organic frameworks (MOFs),^19^ metal nanoparticles,^20^ metal oxide-doped graphene,^21^ and carbon nanotubes (CNTs),^22^ and layered double hydroxides^23,24^ (LDH). Nano catalysts have gained attention due to their unique properties, which enhance the catalytic process.

Cobalt-based salts, mixed metal oxides, and cobalt nanoparticles have been identified as effective catalysts for achieving high yields of BHET. For instance, Chen *et al.* reported that cobalt–aluminium mixed oxides produced a BHET yield of 69%.^25^ Ultra small cobalt nanoparticles were found to function as regenerable catalysts for the precipitation of BHET from PET without the need for external solvents.^20^ Another promising catalyst is CoAlCO_3_-LDH, which demonstrated a remarkable BHET yield of 96%. Cobalt chloride and cobalt-based metal–organic complexes,^5^ CoFe_2_O_4_ and its composites, exhibited good catalytic activity.^26^ Notably, a cobalt-based ionic liquid grafted onto graphene exhibited high catalytic activity, achieving a 95% yield of BHET under mild conditions, along with simple recovery methods.^27^

Cobalt oxide and mixed oxide spinels are reported as glycolysis catalysts by Imran *et al.*^3^ Although cobalt oxide is known to act as a catalyst in glycolysis, none of its reported forms possess inherent magnetic properties that would allow for magnetic separation. The cobalt oxide spinels reported by Imran *et al.* were prepared by precipitation of cobalt hydroxide from corresponding nitrates, followed by calcination at high temperature to get the oxide.

In the present work, magnetic cobalt nanoparticles (CONP) were prepared by three different routes, *viz.*; NaBH_4_ routes,^28^ hydrothermal route,^29^ and combustion routes^30^ and demonstrated as catalysts for the glycolysis of PET. The prepared catalysts were characterized using FTIR, SEM-EDS, surface area analysis, and XRD techniques. PET glycolysis was performed using CONP catalysts prepared by different synthetic routes. The BHET produced was characterized using DSC, HPLC, and NMR techniques. Among the catalysts tested, the CONP catalyst prepared *via* the NaBH_4_ route proved to be the most effective, yielding 97% BHET with just 1% catalyst at a temperature of 180 °C within a reaction time of 2 hours. Additionally, the effects of various reaction conditions, such as temperature, reaction time, and the ratio of PET to EG, were also investigated. The CONP–NaBH_4_ catalyst is magnetically separable from the solution after the reaction and can be reused without losing its effectiveness.

## 2. Experimental materials

Co(NO_3_)_2_·6H_2_O (Alfa Aesar, 98%), CoCl_2_·6H_2_O (Alfa Aesar, 98%), NaBH_4_ powder (Aldrich >98%), ethanol (Absolute, Merck), urea (CO(NH_2_)_2_), sucrose (SD fine chemicals), and ethylene glycol (Alfa Aesar, 99%) were used as such. BHET (Sigma-Aldrich, >98%) was used after recrystallization. PET cut pieces (dimensions ∼1–2 mm) were obtained from waste mineral water bottles. HPLC grade methanol (99.9%, Sigma-Aldrich) was used for HPLC analysis of the glycolyzed products.

### 2.1. Synthesis of cobalt oxide nanoparticles (CONP)

Synthesis by NaBH_4_ route:^28^ magnetic Co_3_O_4_ nanoparticles were synthesized through the following procedure. CoCl_2_·6H_2_O (0.877 g) was added in 30 mL of absolute ethanol while stirring at RT. After complete mixing, NaBH_4_ (1.024 g) was added, and the resulting combination was stirred for 10 min at RT. The obtained material was magnetically separated and then washed completely with warm ethanol and water. The magnetic CONPs were obtained after drying the product at 65 °C for 2 h (CONP–NaBH_4_).

Synthesis by modified solution combustion method:^30^ aqueous solutions of cobalt nitrate hexahydrate (3.305 g) and sucrose (3.805 g) were dissolved in 40 mL of distilled water. The solution mixture was magnetically stirred for 1 h and heated to 350 °C in air. After complete water evaporation, auto ignition started with the release of large amounts of gases. Finally, a fine black product was collected, washed with water, dried, and calcined at 600 °C for 5 h. The final product is labelled as CONP-combustion.

Hydrothermal method:^29^ in a typical synthesis, 1.475 g of cobalt chloride (CoCl_2_) and 0.305 g of urea (CO(NH_2_)_2_) were dissolved in 25 mL of distilled water to form homogeneous solutions, respectively. The urea solution was then added dropwise to the CoCl_2_ solution with stirring. The mixture was then transferred to a 100 mL Teflon-liner autoclave, which was sealed and heated to 105 °C for 6 h. After the autoclave was cooled to room temperature, the resulting pink precipitate was separated by centrifugation, washed three times with distilled water and ethanol, respectively, and dried in an oven at 70 °C. The dried product was then sintered at 300 °C for 3 hours. The black powder obtained is named as CONP-hydrothermal.

**Fig. 1:**
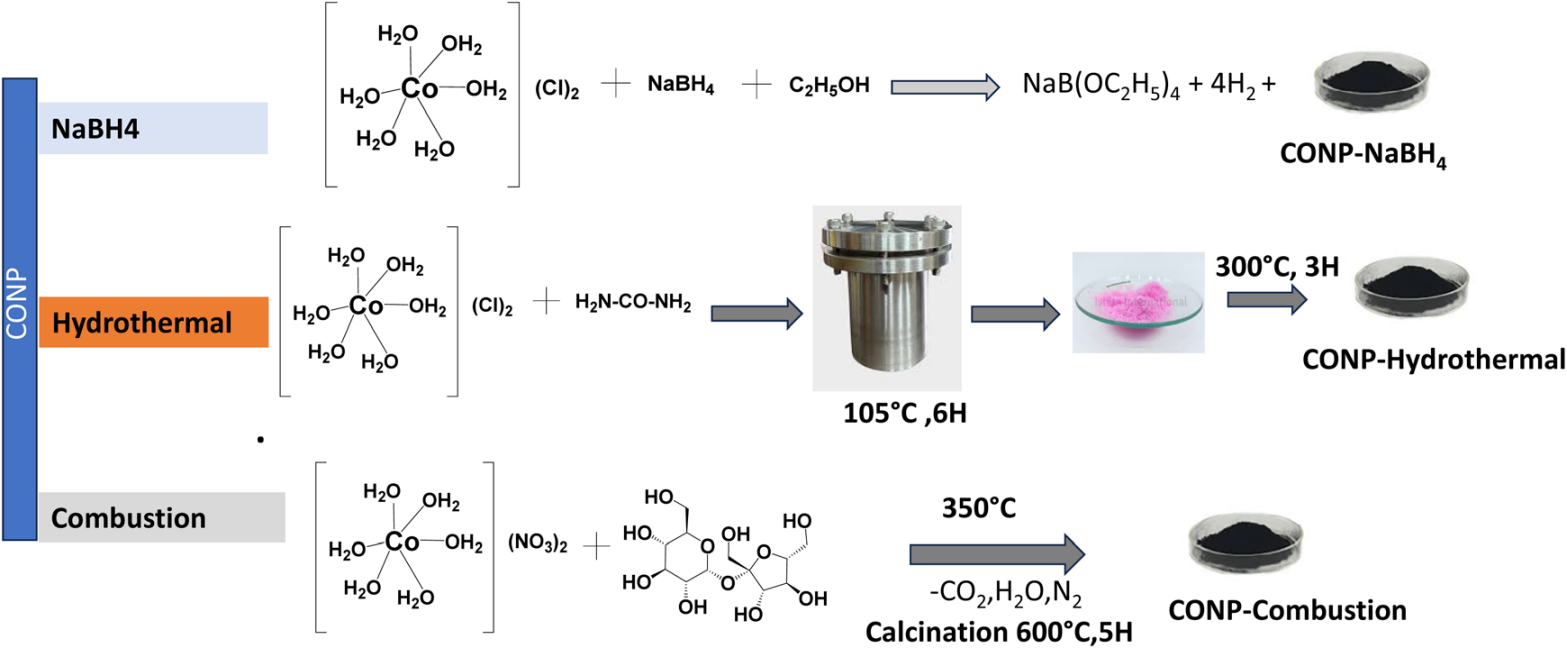
Schematic of catalyst synthesis.

Schematic of the above-mentioned syntheses are shown in Fig. 1.

PET Glycolysis:^16,24^ 1.0 g of PET pieces obtained from mineral water bottles, 0.01 g (1%) CONP, and 10 ml ethylene glycol were heated in a 100 ml two-necked round-bottom flask fitted with a water jacketed condenser at 180 °C for 2 hours with a stirring rate of 400 rpm at ambient pressure. A heating mantle with temperature control was used for the experiment. Temperature of the reaction mixture is also monitored using a thermometer inserted into the reaction mixture. After 2 hours, the temperature was decreased to 100 °C, and un-depolymerized PET that remained in the solution was separated. Then, hot water was added to the system and filtered to separate the catalyst. The filtrate was kept at 4 °C for 12 hours to precipitate BHET. Precipitated BHET was washed with water and dried at 80 °C for 12 hrs. Dried BHET was characterized by FTIR, FTNMR, DSC, and HPLC techniques.

PET conversion was calculated using the following equation.
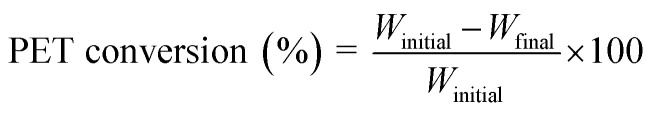
where *W*_initial_ and *W*_final_ are the initial weight of PET and weight of unreacted PET respectively.

The yield of BHET was determined through HPLC analysis. First, the products from the glycolysis reaction were filtered to remove the catalyst and any insoluble oligomers. The resulting filtrate was then diluted to a final volume of 100 ml using HPLC-grade methanol. 100 μL of this solution was further diluted to 10 ml with methanol before injection into the HPLC system. Using a standard BHET, a calibration graph was plotted based on the peak areas obtained from the HPLC analysis. The concentration of BHET in the sample was then estimated using this calibration graph. The yield of BHET was calculated as follows:
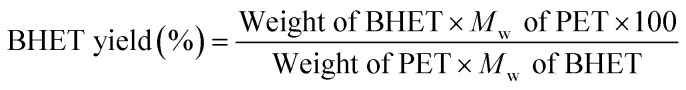
where, *M*_w_ of BHET corresponds to the molecular weight of BHET (254 g mol^−1^) and *M*_w_ of PET is the molecular weight of the PET repeating unit (192 g mol^−1^).

### 2.2. Characterization

FTIR spectra were recorded using a Nicolet iS50 FTIR spectrometer. IR spectra of CONP and BHET were obtained in the region of 400–4000 cm^−1^ by pelletizing the samples with KBr. The IR spectrum of PET was measured using an attenuated total reflectance (ATR) accessory. All spectra were recorded with a resolution of 4 cm^−1^, and 36 scans were accumulated for each. Raman spectra of the CONPs were acquired using a WiTec alpha 300 confocal Raman microscope. Scanning electron microscopy (SEM) imaging analysis was performed with a Carl Zeiss Gemini 500 field emission electron microscope equipped with Bruker detectors. Prior to analysis, the samples were coated with a conductive layer of gold/palladium (80 : 20). Scanning Transmission Electron Microscopic (STEM) analysis was carried out using GeminiSEM 500 (Make: Carl Zeiss). Sample was dispersed in IPA and drop casted on copper grid. Analysis of the dried sample was done using electron beam of accelerating voltage 20V and imaging was done by STEM detector. High resolution transmission electron microscopy (HRTEM) analysis was done using Geol JEM 2100 transmission electron microscope with a 200 kV electron gun.

The BET surface area of the samples was determined using Quantachrom, Novtouch, lLX2. Degassing of the sample was done at 150 °C for 6 hours in flowing nitrogen gas before the sample analysis. Pore size distribution is computed by DFT method. Pore volume is calculated at partial pressure 0.99. The melting point was determined using a TA Instruments 2920 differential scanning calorimeter (DSC). The sample was placed in an aluminum pan and heated from 25 °C to 250 °C at a rate of 5 °C per minute under a nitrogen flow of 10 mL per minute. Both ^13^C and ^1^H nuclear magnetic resonance (NMR) spectra were recorded using a Bruker Avance 400 MHz NMR spectrometer, with CDCl_3_ used as the solvent. X-ray diffraction (XRD) measurements were conducted using a Bruker D8 Discover X-ray diffractometer operating with a copper anode (40 kV, 40 mA). Elemental analysis was performed by inductively coupled plasma atomic emission spectrometry (ICP-AES) using a Thermofisher Scientific iCAP 7400. The magnetic characterization was performed using a Quantum design VersaLab in a field of −30 kOe to 30 kOe at 300K. XPS analysis was caried out with Thermo Fisher Scentific Nexsa G2 surface analysis system. High-performance liquid chromatography (HPLC) analysis was performed using a PerkinElmer UHPLC system (Model: Flexar F-10). Chromatographic separation was achieved employing a Brownlee analytical C8 column (100 mm × 4.6 mm), with methanol serving as the mobile phase. The flow rate was maintained at 1 mL min^−1^ and the injection volume was set to 5 μL. Eluted analytes were detected using a UV detector at a wavelength of 254 nm.

## 3. Results and discussion

### 3.1. Characterization of the catalysts

CONP synthesised using three methods were characterised by FTIR, Raman spectroscopy, XRD, FESEM-EDS, and surface area analysis. EDS of the catalysts revealed the presence of Co, O, and minor amounts of C in the catalysts (Fig. S1–S3).

**Fig. 2:**
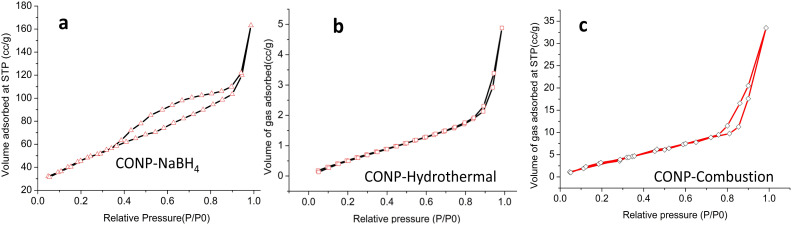
Adsorption–desorption hysteresis curve of CONPs.

Average BET surface areas of the prepared catalysts with standard deviations are listed in Table 1. Surface area is highest for catalyst prepared through sodium borohydride route and lowest for the one prepared through hydrothermal route. Total pore volume calculated by DFT is shown in Table 1. Pore distribution curves are given in Fig. S4. All the three samples are mesoporous with pores size between 2 and 20 nm. Fig. 2a–c show the adsorption–desorption hysteresis loops of CONPs, which are characteristic of mesoporous materials.

**Table 1:** Surface area of the catalysts

	Catalyst	Surface area (m^2^ g^−1^)	Total pore volume (cm^3^ g^−1^)
1	CONP–NaBH_4_	79.1 ± 12.1	0.253
2	CONP-hydrothermal	1.9 ± 0.99	0.008
3	CONP-combustion	29.5 ± 5.8	0.052

**Fig. 3:**
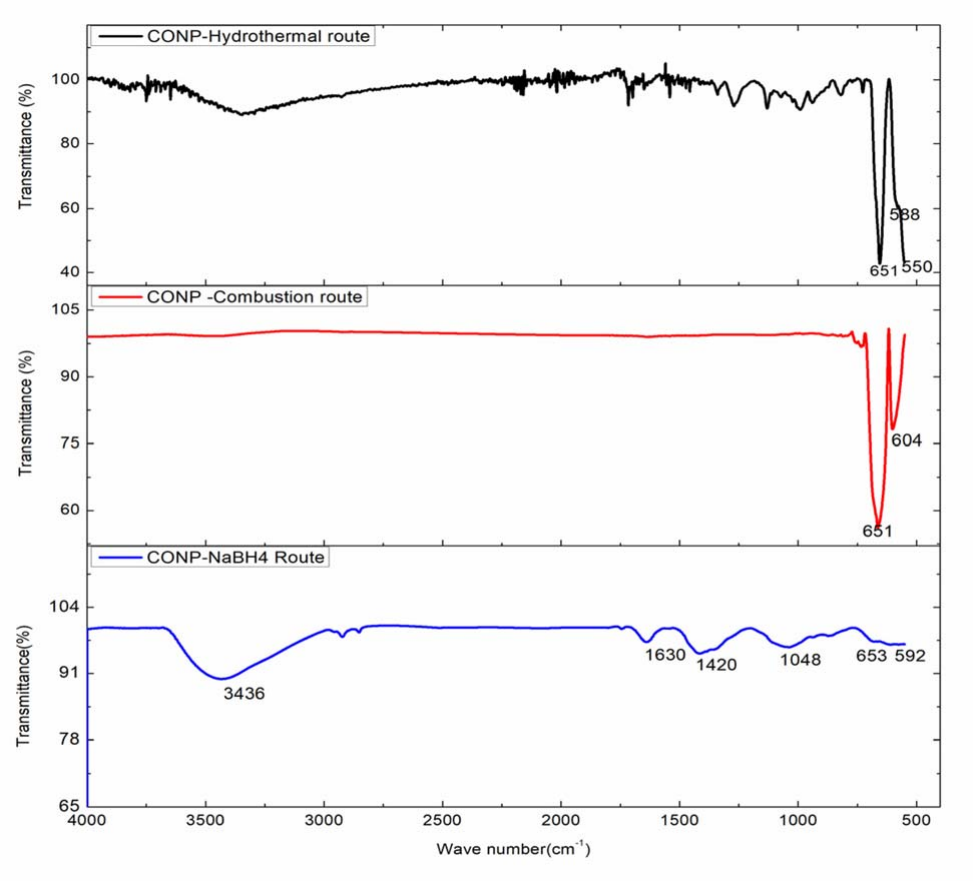
FTIR spectra of the CONP prepared *via* different routes.

Overlaid FTIR spectra of the CONP are shown in Fig. 3. Cobalt oxide prepared *via* hydrothermal and combustion routes showed sharp peaks at 651 cm^−1^ and 600 cm^−1^ due to Co_3_O_4_ spinels. FTIR spectral peak of CONP prepared by borohydride reduction indicates the amorphous nature. In addition to peaks around 650 and 592 cm^−1^, peaks were observed at 1420 and 1048 cm^−1^ due to –OH deformation modes of –CoO(OH).^31^ Borate stretching falls in the range –BO_3_ at 1433 cm^−1^ and –BO_4_ structures at 1102 cm^−1^.^32^ Stretching and bending vibrations of adsorbed moisture are seen around 3400 and 1630 cm^−1^.

**Fig. 4:**
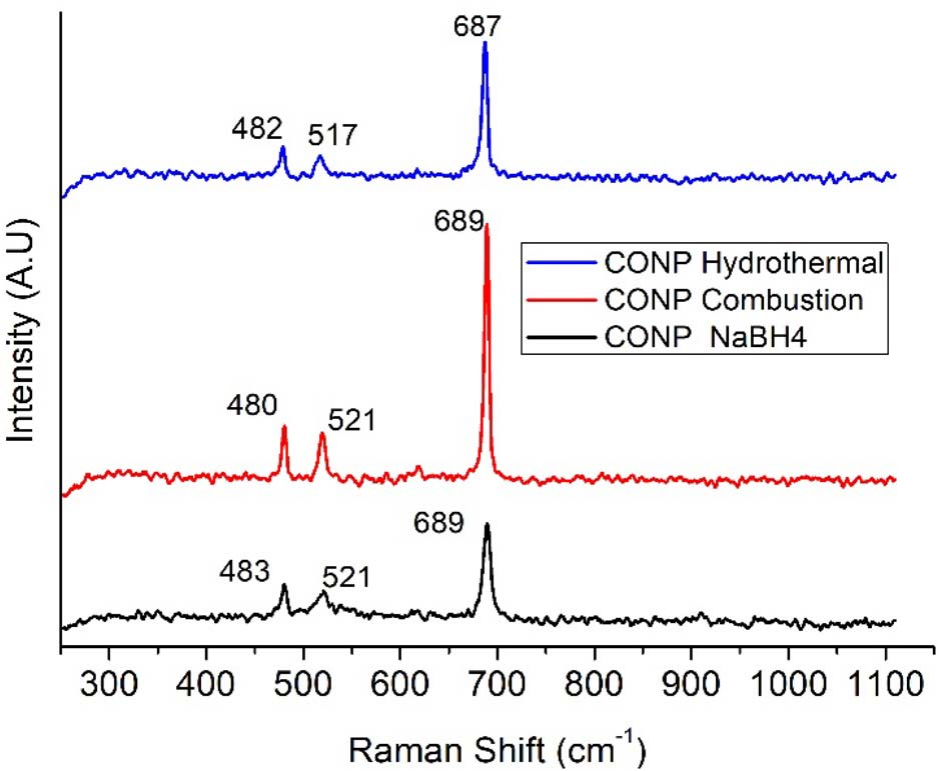
Raman spectra of CONP.

Overlaid Raman spectra of the CONP are shown in Fig. 4. Peaks at, 482, 517 and 687 cm^−1^ correspond to E_g_, F_2g_ and A_1g_ modes of the Co_3_O_4_ respectively.^33–35^ Raman peaks of CONP–NaBH_4_ are broad compared to the other two indicating amorphous nature of the catalyst. Difference in crystallinity of the catalysts are evident from the FWHM of the peak at 687 cm^−1^. FWHM of the peak is 9.1 cm^−1^ for CONP–NaBH_4_, while it is 5.7 and 6.3 cm^−1^ for CONP-combustion and CONP-hydrothermal respectively.

**Fig. 5:**
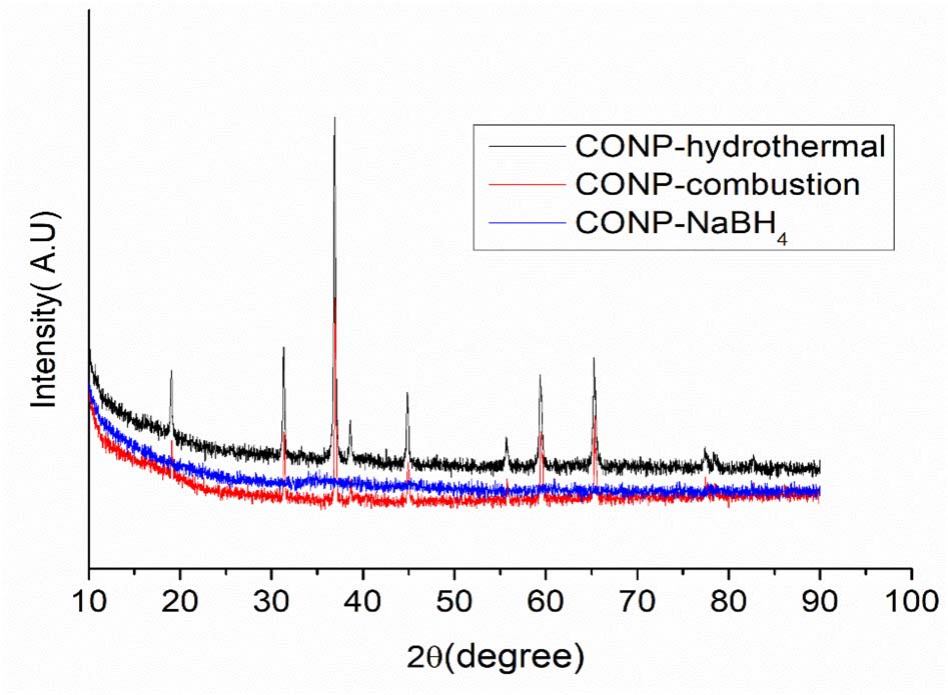
XRD of CONP catalysts.

**Fig. 6:**
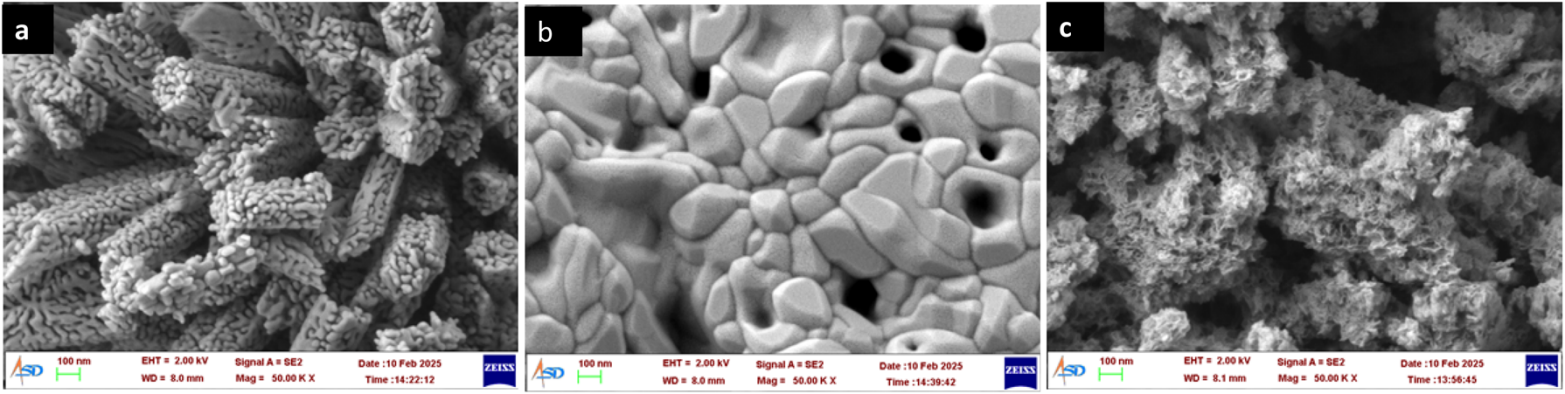
FESEM images of the CONP catalysts prepared (a) CONP hydrothermal, (b) CONP combustion and (c) CONP–NaBH_4_.

XRD patterns of the three catalysts are shown in Fig. 5. The diffraction pattern is characteristic of Cubic Co_3_O_4_ for CONP-hydrothermal and CONP-combustion, while no diffraction pattern obtained for CONP–NaBH_4_ confirming its complete amorphous nature. The surface morphology of the prepared catalysts was studied using FESEM images (Fig. 6). CONP-hydrothermal showed well defined rod-shaped nano aggregates of micrometre length. Size of each particle in aggregate is ≈20 nm. CONP-combustion showed aggregates with particles of nanometre to sub-micrometre size, with micropores in between. SEM image of CONP–NaBH_4_ shows a foam like morphology. SEM image indicates that material is nanoporous.

### 3.2. Magnetic properties of the CONP

Bulk cobalt oxide (Co_3_O_4_) is antiferromagnetic, resulting from antiferromagnetic ordering and overall cancellation of the magnetic moments. However, Co_3_O_4_ nanoparticles may exhibit weak ferromagnetic behaviour at room temperature due to uncompensated surface spins, finite size effects, and structural defects and vacancies. Though CONP-hydrothermal and CONP-combustion are slightly magnetic due to these nano effects, this is not sufficient for magnetic separation from a highly viscous solution of EG and PET decomposition products. On the other hand, CONP–NaBH_4_ is sufficiently ferromagnetic for separation from the reaction mixture. It is not the nano effect alone that contributes to the good magnetic properties of CONP–NaBH_4_. Magnetisation hysteresis plot for CONP–NaBH_4_ (Fig. S4) is characteristic of ferromagnetic material. The maximum magnetization achieved at high magnetic field (*M*_s_) is 1.1 emu g^−1^, and residual magnetization (*M*_r_) is 0.5 emu g^−1^. *M*_s_ and *M*_r_ values reflect moderate magnetic strength, suitable for separation by magnets. The *H*_c_ value (251 Oe) is characteristic of a soft ferromagnetic material.

There are different views among the scientific community about the composition of products formed during hydrolysis/ethanolysis by sodium borohydride in the presence of cobalt chloride. While the evolution of hydrogen is commonly agreed upon, what happens to the cobalt chloride during hydrolysis/alcoholysis is a question of debate. Many studies consider the black precipitate as cobalt borides (Co_*x*_B) and some reports say a mixture of cobalt borides and cobalt metal.^36,37^ Based on our characterization, especially Raman spectroscopy, the black precipitate formed is mainly cobalt oxide. ICP-AES analysis of CONP–NaBH_4_ (Table S1) showed the presence of 4.7% B, 44% cobalt, and 1.7% sodium in the sample. The estimated boron content corresponds to 30% of CoB (calculation S1). Sodium present in the sample suggests that this percentage could be even lower, as some fraction of boron may be present as borate. Borate vibrations are present in the FTIR spectrum of CONP–NaBH_4_, but the peak at 789 cm^−1^ corresponding to the *ν*(Co–B) in cobalt boride is absent in the FTIR spectrum.^38^

XPS analysis of CONP–NaBH_4_ was carried out get further clarity on the structure (Fig. S6). The XPS spectra of Co 2p level shows characteristic binding energies of Co^2+^ and Co^3+^ oxidation states correspond to the presence of Co_3_O_4_, CoO and Co (OH)_2_.^36,37^ B 1s XPS spectrum showed as single peak at 191 eV due the presence of BO_2_^−^ species,^36,39^ O 1s XPS revealed the presence of several non-equivalent states of oxygen atoms. The binding energy values correspond to O^2−^, –OH, O^2−^ spinel, and H_2_O.^40,41^

**Fig. 7:**
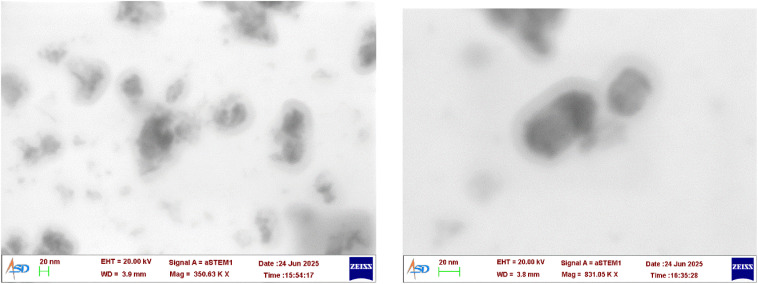
STEM images of CONP–NaBH_4_.

The STEM image of the catalyst revealed a core shell morphology as shown in Fig. 7. The shell thickness is measured as 14–16 nm from the STEM image. Netskina *et al.*^42^ have proposed a core shell structure for amorphous ferromagnetic cobalt–boron composition reduced by sodium borohydride. The shell thickness reported by them is 2–3 nm, but the catalyst reported here shows a shell thickness of 14–16 nm. The higher shell thickness may be the reason for absence of CoB peak in XPS. Similar observation was reported in the XPS of amorphous cobalt borate nanosheet-coated cobalt boride.^43^

**Fig. 8:**
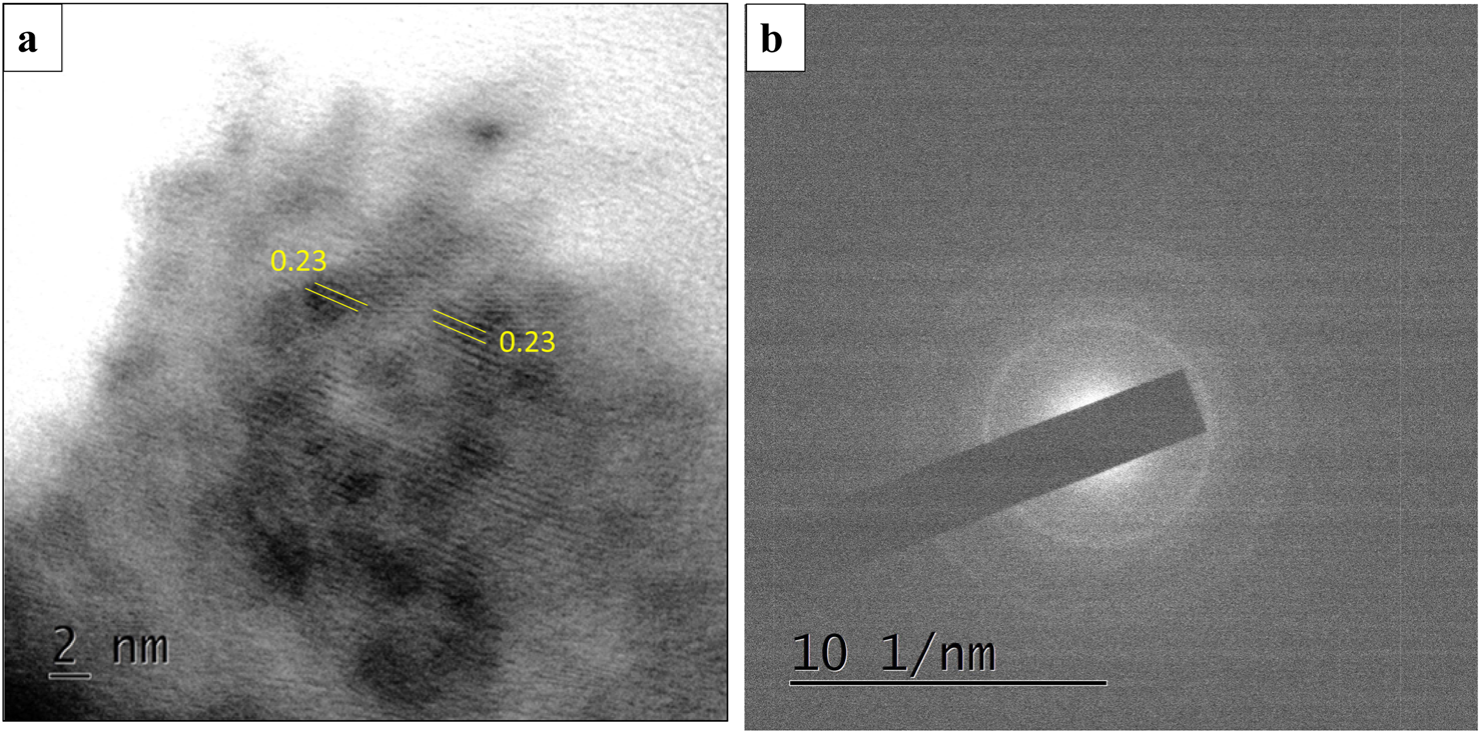
(a) TEM image and (b) SAED pattern of CONP–NaBH_4_.

The HRTEM images of CONP–NaBH_4_ is shown in Fig. 8. Core–shell structure is evident in TEM images also. The core showing nanocrystalline nature with a lattice fringe spacing of 0.23 nm corresponding to d spacing of 101 plane of CoB (JCPDS card 03-0959) and shell is amorphous in nature.^44^ The SAED pattern shows diffuse rings rather than sharp spots, indicating the catalyst is nanocrystalline.

### 3.3. Evaluation of the synthesised CONP as catalysts for glycolysis of PET

**Fig. 9:**
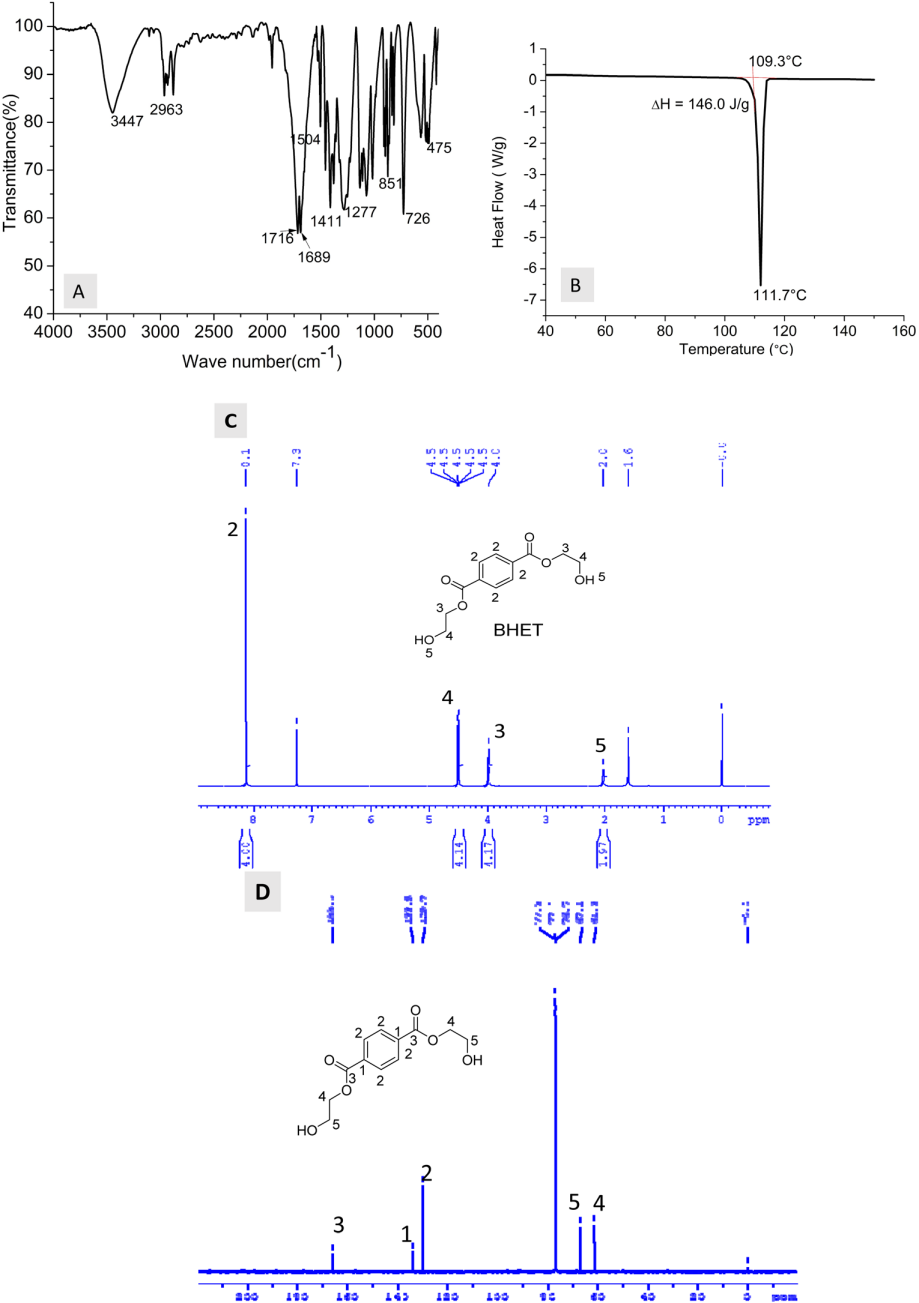
Characterisation of BHET (A) FTIR spectrum, (B) DSC, (C) ^1^H NMR spectrum and (D) ^13^C NMR spectrum.

PET glycolysis experiments were done at 180 °C for 2 hours, with 1% catalyst and PET : EG ratio of 1 : 5. BHET crystallized out from glycolysate was characterized by NMR and FTIR spectroscopy (Fig. 9A, C and D). FTIR spectrum of the separated glycolysis product has characteristic peaks of BHET. DSC of the BHET is shown in Fig. 9B. The purity of the separated BHET is evident from the sharp endotherm, with peak onset at 109 °C due to the melting of BHET. The reported melting point of BHET dimer is between 162 to 166 °C. The obtained melting point ‘109 °C’ is matching with that reported for pure BHET. The peaks and integral values in ^1^H NMR spectrum clearly match with BHET structure. The signals at 61.3, 67.0, 166.0, 133.9, and 129.7 ppm in ^13^C NMR are also characteristic of the BHET monomer. Non-appearance of peak at 63 ppm in ^13^C NMR and 5 ppm in ^1^H NMR confirms the absence of dimer. It is established from NMR analysis that the separated BHET is 100% pure as the spectra do not show any additional peak.

**Fig. 10:**
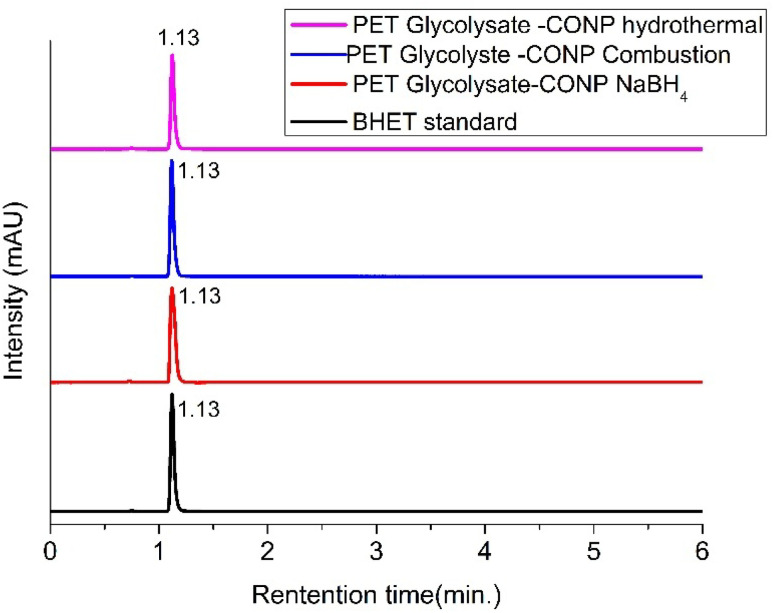
Overlaid HPLC of PET glycolysates obtained using different CONP catalysts and BHET standard solution.

The glycolysate was analysed by HPLC to find out the BHET yield. Calibration graph for estimation of BHET yield is given in Fig. S7. Overlaid chromatograms of BHET standard and the PET glycolysates are shown in Fig. 10. Peak at 1.1 minutes is due to BHET, and no dimer peak (which is expected around 1.34 minutes) is present in any of the chromatograms.

**Fig. 11:**
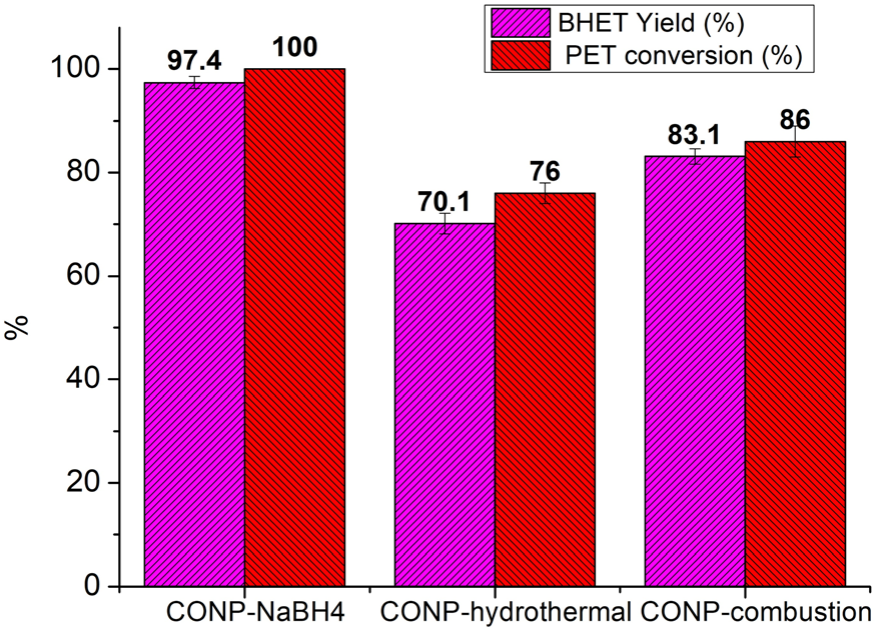
BHET yield and PET conversion obtained using CONP catalysts (experimental conditions: reaction time 2H, catalyst 1%, EG/PET 10, temperature 180 °C).

The PET conversion and BHET yield obtained with 1% of the CONP catalysts at an EG/PET ratio of 10, and a reaction temperature of 180 °C for 2 hours are given in the Fig. 11.

Among the catalysts prepared, the CONP synthesized using the sodium borohydride route demonstrated the best performance, achieving 97% yield of BHET and 100% conversion of PET. The second-best performer was the CONP-combustion method, which resulted in 83% yield of BHET and 86% conversion of PET. In contrast, performance of CONP produced *via* the hydrothermal route was the lowest, yielding only 70% BHET and 76% conversion of PET. Mass balance summary for glycolysis reactions with three catalysts is given in Table S3.

### 3.4. Better efficiency of CONP–NaBH_4_ catalyst

Among the prepared catalysts, CONP–NaBH_4_ is amorphous, while the other two, which involve calcination step, and are composed of crystalline Co_3_O_4_ for which surface area and porosity are low compared to the amorphous. The porous nature of the CONP–NaBH_4_ catalyst contributes to its higher active surface area. Though all three samples are mesoporous, the total pore volume for CONP–NaBH_4_ is 0.25 cm^3^ g^−1^ against 0.05 cm^3^ g^−1^ and 0.008 cm^3^ g^−1^, respectively, for CONP-combustion and hydrothermal. Also, the presence of surface hydroxyl and oxyhydroxy groups in CONP–NaBH_4_ enhances catalytic activity by promoting the deprotonation of EG.

This is the first report on magnetically separable CONP prepared by NaBH_4_ reduction route for PET glycolysis. Amorphous ferromagnetic cobalt–boron composition reduced by sodium borohydride was studied widely as hydrogen generation catalysts,^36,37,42^ but their use as transesterification catalyst for PET has not explored yet. Though NaBH_4_ reduction was reported to make ultrasmall cobalt nanoparticles for PET glycolysis, their synthesis method is different^20^ and magnetic separation was not attempted. Performance of CONP–NaBH_4_ is in parr with the reported cobalt-based catalysts for PET glycolysis (Table 2). The highest BHET yield reported among cobalt based catalyst for PET glycolysis 99% is for CoAl LDH@Fe_3_O_4_.^23^ But CoAl LDH is not inherently magnetic and magnetic support is used to make it magnetically regenerable.

**Table 2:** Comparison cobalt based catalyst for PET glycolysis^a^

	Catalyst	BHET %	EG/PET	Time (h)	Catalyst (%)	Glycolysis temperature	Regeneration
1	CoAl mixed oxides^25^	69	5	0.83	1	196	Not reported
2	Ultrasmall cobalt nanoparticles^20^	77	20	3	1.5	180	Regenerable by filtration
3	CoFe_2_O_4_ (ref. 46)	77	6	6	4	190	Magnetic regeneration
4	CoFe_2_O_4_@ZIF-8/ZIF-67 ^46^	84	5	1	1	200	
5	CoFe_2_O_4_/C10-OAC^46^	95	5	2.5	2	195	
6	CoCl_2_ (anh)^5^	65	10	3	1	190	Not reported
7	CoCl_2_ (anh)/dcype^5^	71					
8	CoCl_2_ (anh)/dppe^5^	53					
9	CoCl_2_ (anh)/dppf^5^	31					
10	CoAlCO_3_-LDH@ Fe_3_O_4_ (ref. 23)	99	10	2	1	180	Magnetic regeneration
11	Cobalt-based ionic liquid grafted on graphene^27^	95				190	
12	Cobalt oxides recycled from spent batteries^47^	10	8	2	1	196	Not reported
13	Cobalt oxide–NaBH_4_ reduction (present work)	97	5	2	1	180	Magnetic regeneration

a BHET yield under optimum conditions are compared.

Turnover Frequency (TOF) is a measure of catalytic activity and it represents the number of reactant molecules converted to product per active site on the catalyst per unit time. The TOF calculated for CONP–NaBH_4_ is 271 h^−1^, which is higher than reported for the commercial glycolysis catalyst Zn (OAc)_2_ ^45^ (calculation S2).

Hydrogen gas is evolved as a byproduct in the preparation of CONP–NaBH_4_. Cobalt chloride is a well-established catalyst for hydrogen generation from NaBH_4_ through hydrolysis or alcoholysis reactions, and the catalyst CONP–NaBH_4_ could also be produced in the future as a valuable byproduct during hydrogen production. This approach presents an opportunity to combine clean energy generation with the principles of a circular economy for plastic manufacturing, aligning resource efficiency with sustainability goals.

Environmental footprint for the synthesis of CONP–NaBH_4_ is low compared to hydrothermal or combustion routes. Combustion routes and hydrothermal routes require temperatures up to 600 °C and 300 °C, respectively. Greenhouse gas (carbon dioxide) evolution is another drawback of hydrothermal and combustion routes.

### 3.5. Optimisation of the reaction conditions

**Fig. 12:**
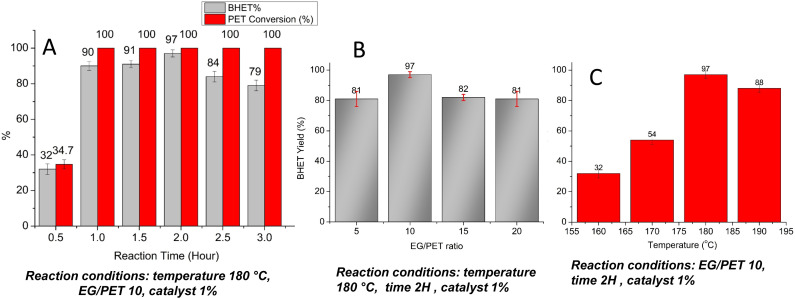
Effect of (a) reaction time (b) EG/PET ratio and (c) reaction temperature on BHET yield.

The effect of different reaction parameters on BHET yield, such as EG/PET ratio, temperature, and reaction time, were studied (Fig. 12). The reaction time was varied from 0.5 hours to 3 hours, while other parameters remained constant: temperature was set at 180 °C, catalyst concentration 1%, and the EG/PET ratio 10. Yield of BHET increases with reaction time, reaching a maximum of 97% at 2 hours, after which it begins to decline (Fig. 12A). It is also noteworthy that 90% of the BHET yield can be achieved in a short reaction time of just 1 hour. The decrease in BHET yield after 2 hours may be attributed to the re-polymerization of BHET.

EG/PET ratio was optimized by varying the ratio from 5 to 20 (Fig. 12B). Temperature, reaction time, and catalyst concentrations were fixed as 180 °C, 2 hours, and 1% respectively. BHET yield increased with the increase in EG/PET ratio and reached a maximum at a ratio of 10. Further increase in EG decreases the BHET yield. A similar trend is reported for cobalt aluminium LDH catalyst.^23^

Glycolysis increases with rising temperatures, reaching an optimal value before declining (Fig. 12C). The optimal reaction temperature for CONP–NaBH_4_ was identified as 180 °C; however, further increasing the temperature to 190 °C resulted in a decrease in BHET yield to 88%. It has been reported that high reaction temperatures may also promote the polymerization of the BHET formed.^48^

### 3.6. Regeneration of the catalyst

**Fig. 13:**
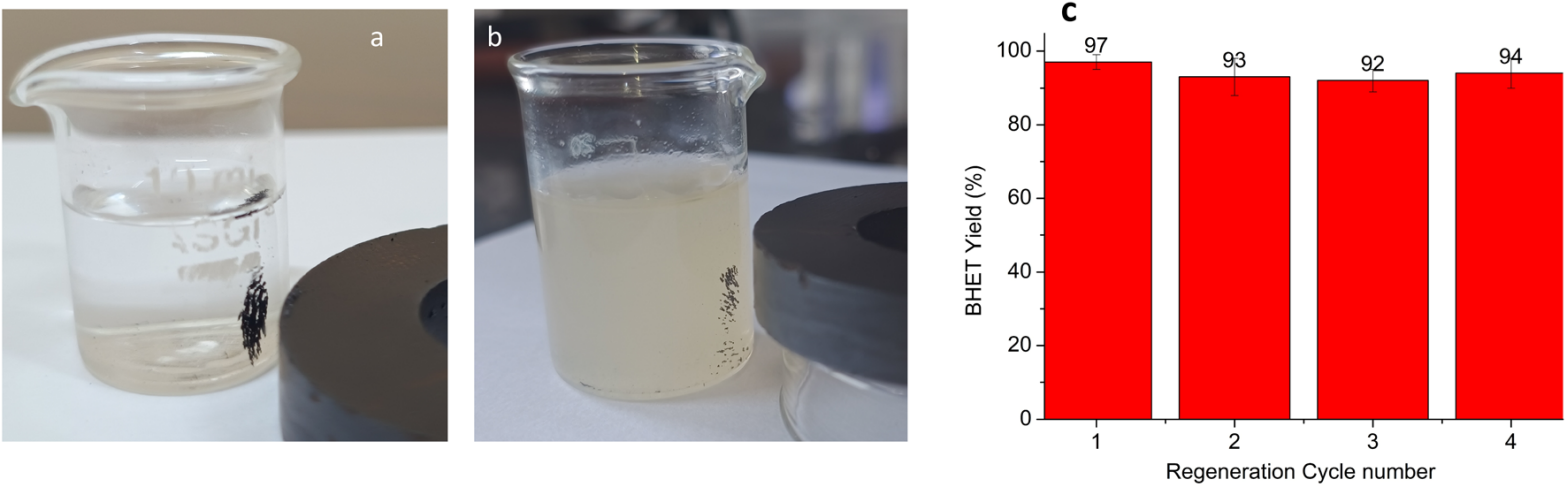
(a) and (b) Magnetic separation of the catalyst from EG and from the reaction mixture after completion of glycolysis and (c) BHET yield during four regeneration cycles.

Reusability is a favoured characteristic for sustainable catalysts. CONP is inherently magnetic in nature, and the magnetic property is utilised for its regeneration from the glycolysis mixture. Fig. 13 shows the magnetic separation of CONP from EG and the reaction mixture after glycolysis. The separated catalyst was dried and used as such for the next cycle. Four cycles of regeneration were carried out successfully without a considerable drop in BHET yield. As the catalyst is inherently magnetic, it does not require any external magnetic support to make it suitable for magnetic separation.

**Fig. 14:**
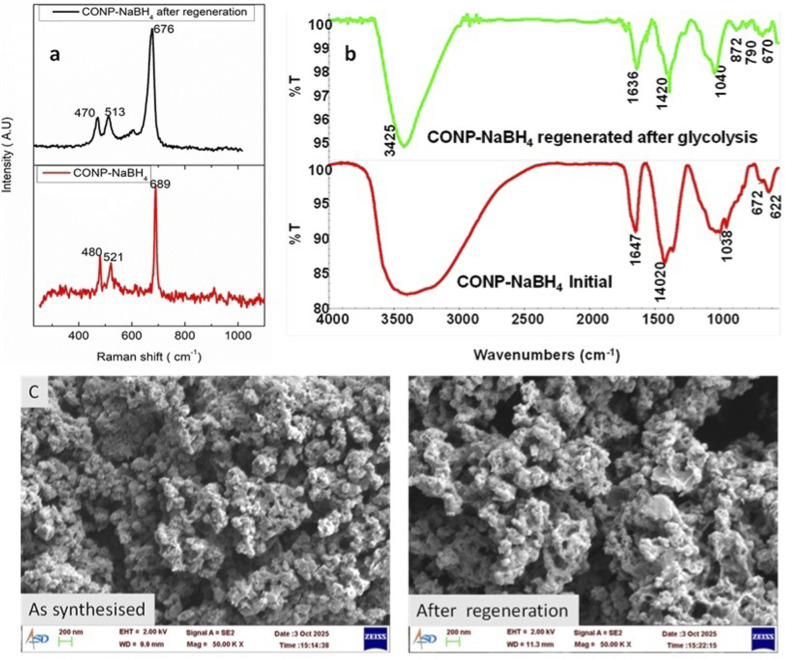
Overlaid (a) Raman spectrum, (b) FTIR spectrum and (c) SEM image of CONP–NaBH_4_ as such and after regeneration.

Overlaid Raman spectra of the fresh catalyst and the catalyst separated after regeneration are shown in Fig. 14a. Peak postions are corresponding to F_2g_, E_g_, and A_1g_ phonon modes of Co_3_O_4_.^34,35^ Peak broadening and peak red shift are noticed compared to the spectrum before recycling. FWHM of the peak at 678 cm^−1^, was 9.1 cm^−1^ before regeneration, while it increased to 24.5 cm^−1^ for the regenerated catalyst. Increase in FWHM points towards the dissociation of nanoaggregates to smaller nanoparticles after glycolysis. Raman spectra of the catalyst were acquired after each cycle (Fig. S9) and the spectra are characteristic of cobalt oxide.

FTIR spectrum of the catalyst before glycolysis and after glycolysis is shown Fig. 14b. Catalyst after glycolysis also showed peaks corresponding to borate and cobalt oxide, like that of the initial catalyst. A small peak at 790 cm^−1^, which was not seen initially, is evident in the catalyst after glycolysis. As per the literature, this could be due to the Co–B stretching. This peak was not visible in the initial phase may be due to overlapping with borate peaks. SEM images of the catalyst confirm that the regenerated catalyst retains its morphology after reaction. EDS analysis of the regenerated catalyst showed a decrease in sodium compared to the original may be due to the dissolution of soluble sodium borate during reaction and washing. XPS analysis of the regenerated catalyst shows a spectrum similar to the original, but boron content is reduced after glycolysis, confirming the EDS inferences.

ICP-AES analysis of the filtrates from each step was performed to evaluate catalyst leaching into the solution. Cobalt concentration was consistently below 20 ppm (Table S3), confirming minimal leaching and demonstrating the effective separation of the catalyst. In addition, ICP-AES analysis of the recovered BHET failed to detect any cobalt content.

The hot filtration test was carried out to verify heterogeneous catalysis. Details of the test and calculation are given in SI (Tables S4 and S5). After 1 hour, the catalyst was filtered from the reaction mixture, and the reaction was allowed to proceed for another hour without the catalyst. The BHET yield after 2 hours dropped to 84%, compared to 98% in the presence of catalyst, confirming the heterogeneous catalytic pathway.

## 4. Conclusion

CONP catalysts were prepared using three different synthesis methods and characterized. The assessment of these catalysts for glycolysis of PET revealed that those prepared through the sodium borohydride reduction method demonstrated greater activity compared to the other two catalysts made *via* hydrothermal and combustion methods. This is the first report on the use of CONP prepared through NaBH_4_ reduction route for PET glycolysis. The higher activity of the CONP–NaBH_4_ catalyst is attributed to its porous nature and comparatively high surface area. Additionally, the presence of surface hydroxy groups in the catalysts enhances the catalytic activity. A 100% conversion of PET with 97% yield of BHET was achieved using 1% catalyst at 180 °C within a reaction time of 2 hours. The catalyst is magnetically recoverable, and four cycles of regeneration were demonstrated without considerable loss in efficiency. A core–shell structure with ferromagnetic cobalt boride core and CONP shell is proposed for the catalyst. Core is responsible for the magnetic properties, while the catalytic activity can be attributed to the shell. After the fourth cycle, the catalyst shows no signs of chemical degradation, with catalyst leaching remaining below 20 ppm each time. Additionally, the precipitated BHET is free of any catalyst residues. With its straightforward synthesis method, high BHET yield achieved at moderately low temperatures, short reaction times, and excellent recyclability, CONP–NaBH_4_ stands out as a highly promising catalyst for industrial applications. Furthermore, the potential to integrate hydrogen production with CONP–NaBH_4_ synthesis presents an exciting avenue for future development.

## Author contributions

The manuscript was written through contributions of all authors. All authors have given approval to the final version of the manuscript.

## Conflicts of interest

There are no conflicts of interest to declare.

## Supplementary Material

RA-015-D5RA05618G-s001

## Data Availability

Data underlying in this study are available in the article and its online supplementary information (SI). Supplementary Information: EDS of CONP–NaBH_4_, CONP-hydrothermal, and CONP-combustion, magnetisation hysteresis curve, ICP-AES analysis CONP–NaBH_4_, calculation of CoB %, XPS of CONP–NaBH_4_, calibration graph for HPLC, mass balance summary, calculation of TOF, magnetic separation of catalyst from EG, Raman spectra of regenerated catalysts, leaching out of cobalt in each regeneration cycle, and hot filtration test. See DOI: https://doi.org/10.1039/d5ra05618g.

## References

[cit1] Francis R. , Recycling of Polymers: Methods, Characterization and Applications, John Wiley & Sons, 2016

[cit2] Aguado J. and Serrano D. P., Feedstock Recycling of Plastic Wastes, Royal society of chemistry, 2007

[cit3] Imran M., Al-Masry W. A., Mahmood A., Hassan A., Haider S., Ramay S. M. (2013). Manganese-, cobalt-, and zinc-based mixed-oxide spinels as novel catalysts for the chemical recycling of poly (ethylene terephthalate) via glycolysis. Polym. Degrad. Stab..

[cit4] Bartolome L., Imran M., Lee K. G., Sangalang A., Ahn J. K. (2014). Superparamagnetic γ-Fe 2 O 3 nanoparticles as an easily recoverable catalyst for the chemical recycling of PET. Green Chem..

[cit5] Esquer R., García J. J. (2019). Metal-catalysed Poly (Ethylene) terephthalate and polyurethane degradations by glycolysis. J. Organomet. Chem..

[cit6] Imran M., Lee K. G., Imtiaz Q., Kim B.-k., Han M., Cho B. G., Kim D. H. (2011). Metal-oxide-doped silica nanoparticles for the catalytic glycolysis of polyethylene terephthalate. J. Nanosci. Nanotechnol..

[cit7] Marullo S., Rizzo C., Dintcheva N. T., D'Anna F. (2021). Amino acid-based cholinium ionic liquids as sustainable catalysts for PET depolymerization. ACS Sustain. Chem. Eng..

[cit8] Al-Sabagh A., Yehia F., Eissa A., Moustafa M., Eshaq G., Rabie A., ElMetwally A. (2014). Cu-and Zn-acetate-containing ionic liquids as catalysts for the glycolysis of poly (ethylene terephthalate). Polym. Degrad. Stab..

[cit9] Wang H., Li Z., Liu Y., Zhang X., Zhang S. (2009). Degradation of poly (ethylene terephthalate) using ionic liquids. Green Chem..

[cit10] Wang Q., Geng Y., Lu X., Zhang S. (2015). First-row transition metal-containing ionic liquids as highly active catalysts for the glycolysis of poly (ethylene terephthalate)(PET). ACS Sustain. Chem. Eng..

[cit11] Wang Q., Yao X., Geng Y., Zhou Q., Lu X., Zhang S. (2015). Deep eutectic solvents as highly active catalysts for the fast and mild glycolysis of poly (ethylene terephthalate)(PET). Green Chem..

[cit12] Rollo M., Raffi F., Rossi E., Tiecco M., Martinelli E., Ciancaleoni G. (2023). Depolymerization of polyethylene terephthalate (PET) under mild conditions by Lewis/Brønsted acidic deep eutectic solvents. Chem. Eng. J..

[cit13] Liu B., Fu W., Lu X., Zhou Q., Zhang S. (2018). Lewis acid–base synergistic catalysis for polyethylene terephthalate degradation by 1, 3-Dimethylurea/Zn (OAc) 2 deep eutectic solvent. ACS Sustain. Chem. Eng..

[cit14] Wang Z., Jin Y., Wang Y., Tang Z., Wang S., Xiao G., Su H. (2022). Cyanamide as a highly efficient organocatalyst for the glycolysis recycling of PET. ACS Sustain. Chem. Eng..

[cit15] Wang Q., Yao X., Tang S., Lu X., Zhang X., Zhang S. (2012). Urea as an efficient and reusable catalyst for the glycolysis of poly (ethylene terephthalate) wastes and the role of hydrogen bond in this process. Green Chem..

[cit16] López-Fonseca R., Duque-Ingunza I., de Rivas B., Flores-Giraldo L., Gutiérrez-Ortiz J. I. (2011). Kinetics of catalytic glycolysis of PET wastes with sodium carbonate. Chem. Eng. J..

[cit17] Wang S., Wang C., Wang H., Chen X., Wang S. (2015). Sodium titanium tris (glycolate) as a catalyst for the chemical recycling of poly (ethylene terephthalate) via glycolysis and repolycondensation. Polym. Degrad. Stab..

[cit18] Pavel O. D., Tichit D., Marcu I.-C. (2012). Acido-basic and catalytic properties of transition-metal containing Mg–Al hydrotalcites and their corresponding mixed oxides. Appl. Clay Sci..

[cit19] Suo Q., Zi J., Bai Z., Qi S. (2017). The glycolysis of poly (ethylene terephthalate) promoted by metal organic framework (MOF) catalysts. Catal. Lett..

[cit20] Veregue F. R., Pereira da Silva C. T., Moisés M. P., Meneguin J. G., Guilherme M. R. r., Arroyo P. A., Favaro S. L., Radovanovic E., Girotto E. M., Rinaldi A. W. (2018). Ultrasmall cobalt nanoparticles as a catalyst for PET glycolysis: a green protocol for pure hydroxyethyl terephthalate precipitation without water. ACS Sustain. Chem. Eng..

[cit21] Nabid M. R., Bide Y., Fereidouni N., Etemadi B. (2017). Maghemite/nitrogen-doped graphene hybrid material as a reusable bifunctional catalyst for glycolysis of polyethylene terephthalate. Polym. Degrad. Stab..

[cit22] Al-Sabagh A., Yehia F., Harding D. R., Eshaq G., ElMetwally A. (2016). Fe 3 O 4-boosted MWCNT as an efficient sustainable catalyst for PET glycolysis. Green Chem..

[cit23] Thomas D., Ranjan R., George B. K. (2023). Co-Al-CO 3 layered double hydroxide: an efficient and regenerable catalyst for glycolysis of polyethylene terephthalate. RSC Sustainability.

[cit24] Eshaq G., ElMetwally A. (2016). (Mg–Zn)–Al layered double hydroxide as a regenerable catalyst for the catalytic glycolysis of polyethylene terephthalate. J. Mol. Liq..

[cit25] Chen F., Yang F., Wang G., Li W. (2014). Calcined Zn/Al hydrotalcites as solid base catalysts for glycolysis of poly (ethylene terephthalate). J. Appl. Polym. Sci..

[cit26] Krisbiantoro P. A., Chiao Y.-W., Liao W., Sun J.-P., Tsutsumi D., Yamamoto H., Kamiya Y., Wu K. C.-W. (2022). Catalytic glycolysis of polyethylene terephthalate (PET) by solvent-free mechanochemically synthesized MFe2O4 (M= Co, Ni, Cu and Zn) spinel. Chem. Eng. J..

[cit27] Najafi-Shoa S., Barikani M., Ehsani M., Ghaffari M. (2021). Cobalt-based ionic liquid grafted on graphene as a heterogeneous catalyst for poly (ethylene terephthalate) glycolysis. Polym. Degrad. Stab..

[cit28] Ardeshirfard H., Elhamifar D. (2023). An efficient method for the preparation of magnetic Co3O4 nanoparticles and the study of their catalytic application. Front. Catal..

[cit29] Wang G., Shen X., Horvat J., Wang B., Liu H., Wexler D., Yao J. (2009). Hydrothermal synthesis and optical, magnetic, and supercapacitance properties of nanoporous cobalt oxide nanorods. J. Phys. Chem. C..

[cit30] Mohanta J., Dey B., Dey S. (2020). Magnetic cobalt oxide nanoparticles: sucrose-assisted self-sustained combustion synthesis, characterization, and efficient removal of malachite green from water. J. Chem. Eng. Data.

[cit31] Yang J., Liu H., Martens W. N., Frost R. L. (2010). Synthesis and characterization of cobalt hydroxide, cobalt oxyhydroxide, and cobalt oxide nanodiscs. J. Phys. Chem. C..

[cit32] Zhang C. C., Gao X., Yilmaz B. (2020). Development of FTIR spectroscopy methodology for characterization of boron species in FCC catalysts. Catalysts.

[cit33] Tang C.-W., Wang C.-B., Chien S.-H. (2008). Characterization of cobalt oxides studied by FT-IR, Raman, TPR and TG-MS. Thermochim. Acta.

[cit34] Hadjiev V., Iliev M., Vergilov I. (1988). The raman spectra of Co3O4. J. Phys. C: Solid State Phys..

[cit35] Rashad M., Rüsing M., Berth G., Lischka K., Pawlis A. (2013). CuO and Co3O4 nanoparticles: synthesis, characterizations, and Raman spectroscopy. J. Nanomater..

[cit36] Demirci U. B., Miele P. (2010). Cobalt in NaBH4 hydrolysis. Phys. Chem. Chem. Phys..

[cit37] Netskina O. V., Kochubey D. I., Prosvirin I. P., Malykhin S. E., Komova O. V., Kanazhevskiy V. V., Chukalkin Y. G., Bobrovskii V. I., Kellerman D. G., Ishchenko A. V., Simagina V. I. (2017). Cobalt-boron catalyst for NaBH4 hydrolysis: The state of the active component forming from cobalt chloride in a reaction medium. Mol. Catal..

[cit38] Malinina E., Goeva L., Buzanov G., Avdeeva V., Efimov N., Kozerozhets I., Kuznetsov N. (2019). Synthesis and physicochemical properties of binary cobalt (II) borides. Thermal reduction of precursor complexes [CoL n][B 10 H 10](L= H 2 O, n= 6; N 2 H 4, n= 3). Russ. J. Inorg. Chem..

[cit39] Kukula P., Gabova V., Koprivova K., Trtik P. (2007). Selective hydrogenation of unsaturated nitriles to unsaturated amines over amorphous CoB and NiB alloys doped with chromium. Catal. Today.

[cit40] Foelske A., Strehblow H. H. (2000). Passivity of cobalt in borate buffer at pH 9.3 studied by x-ray photoelectron spectroscopy. Surf. Interface Anal..

[cit41] Jena A., Penki T. R., Munichandraiah N., Shivashankar S. (2016). Flower-like porous cobalt (II) monoxide nanostructures as anode material for Li-ion batteries. J. Electroanal. Chem..

[cit42] Netskina O., Kellerman D., Ishchenko A., Komova O., Simagina V. (2018). Amorphous ferromagnetic cobalt-boron composition reduced by sodium borohydride: Phase transformation at heat-treatment and its influence on the catalytic properties. Colloids Surf., A.

[cit43] Tan T., Han P., Cong H., Cheng G., Luo W. (2019). An Amorphous Cobalt Borate Nanosheet-Coated Cobalt Boride Hybrid for Highly Efficient Alkaline Water Oxidation Reaction. ACS Sustain. Chem. Eng..

[cit44] Jose V., Nsanzimana J. M. V., Hu H., Choi J., Wang X., Lee J. M. (2021). Highly efficient oxygen reduction reaction activity of N-doped carbon–cobalt boride heterointerfaces. Adv. Energy Mater..

[cit45] Wu Y., Tang X., Qian S., Huang H., Che Y., Hu Q., Sun M., Cheng Y., Niu Z. (2025). Efficient chemical recycling of mixed plastics by intramolecular glycolysis.. AIChE J..

[cit46] Wang T., Zheng Y., Yu G., Chen X. (2021). Glycolysis of polyethylene terephthalate: Magnetic nanoparticle CoFe2O4 catalyst modified using ionic liquid as surfactant. Eur. Polym. J..

[cit47] Fuentes C. A., Gallegos M. V., García J. R., Sambeth J., Peluso M. A. (2019). Catalytic glycolysis of poly (ethylene terephthalate) using zinc and cobalt oxides recycled from spent batteries. Waste Biomass Valori..

[cit48] Wang H., Yan R., Li Z., Zhang X., Zhang S. (2010). Fe-containing magnetic ionic liquid as an effective catalyst for the glycolysis of poly (ethylene terephthalate). Catal. Commun..

