# Supplementary material for: Magnetically recoverable cobalt oxide nanoparticle catalyst for PET glycolysis
Source: RSC Adv. 2025 Nov 5;15(50):42956–67. doi: 10.1039/d5ra05618g (PMC12587479; doi:10.1039/d5ra05618g)
Supplement: RA-015-D5RA05618G-s001 [file RA-015-D5RA05618G-s001.pdf]

This journal is © The Royal Society of Chemistry 2025

Supporting information

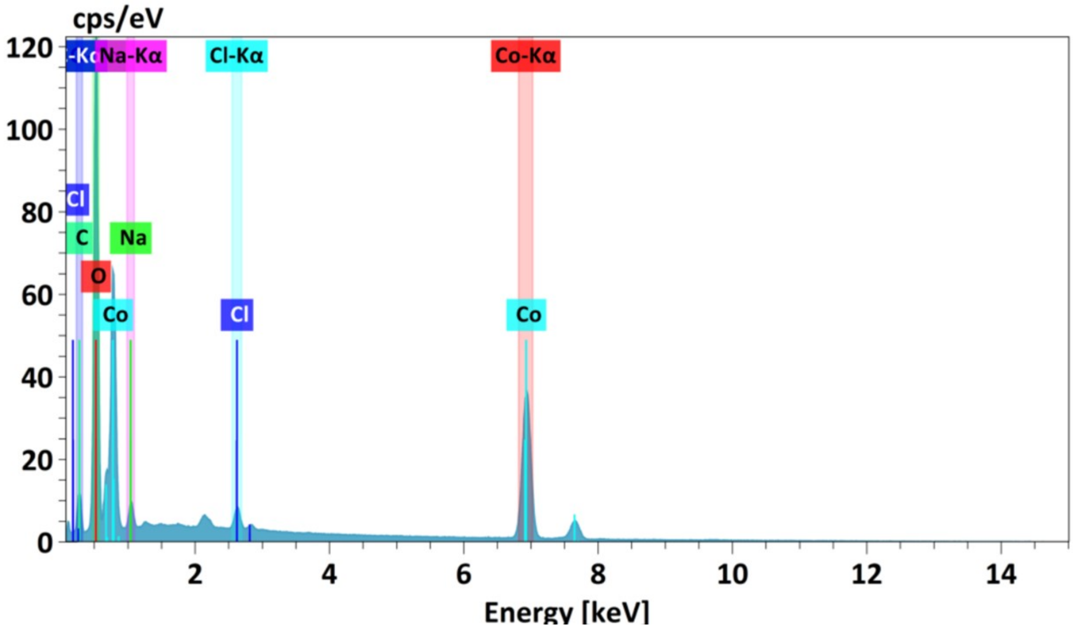

Figure S1. EDS of CONP-$NaBH_4$

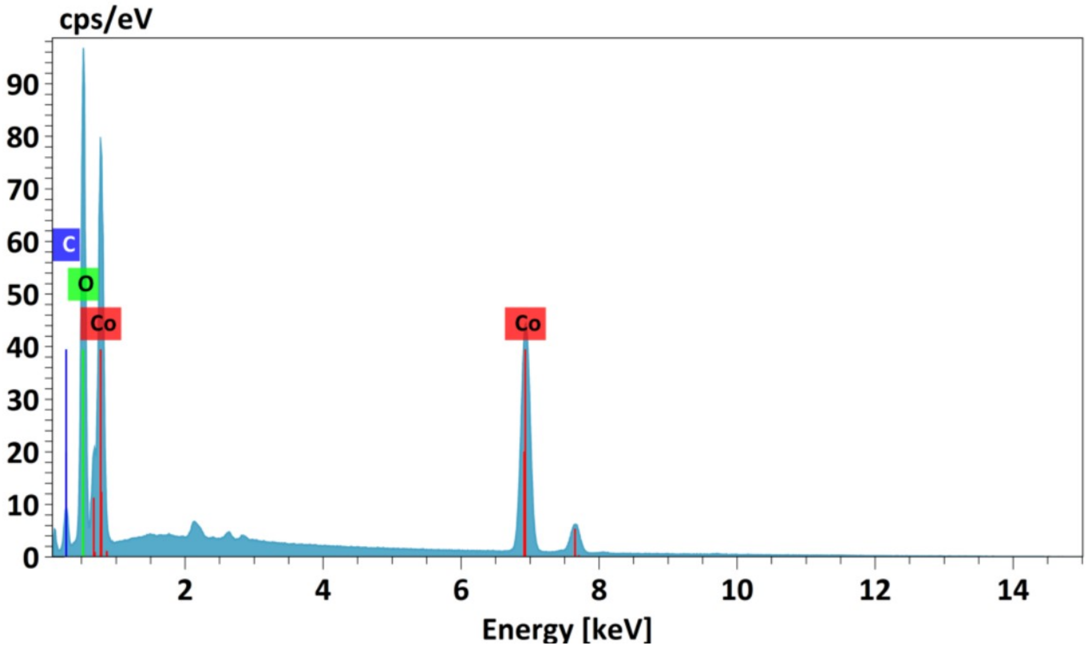

Figure S2. EDS of CONP-Hydrothermal

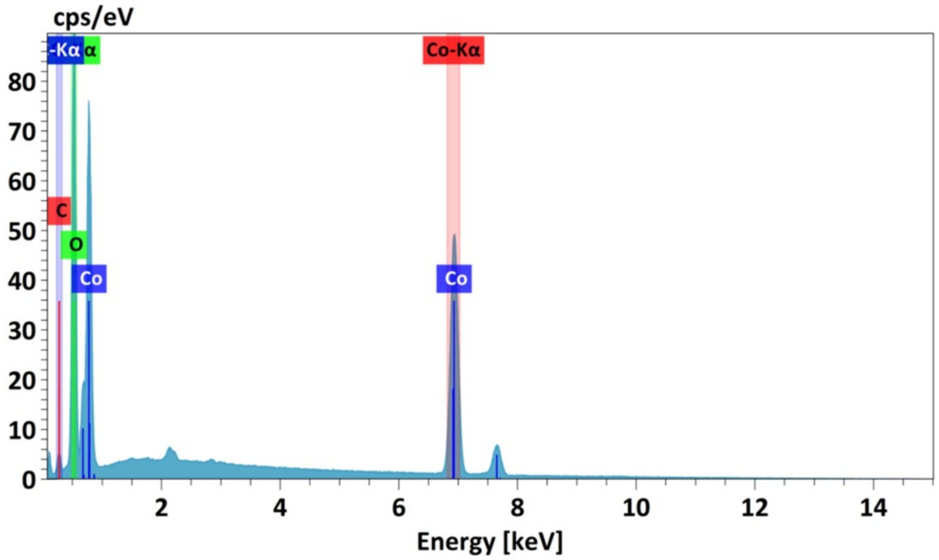

Figure S3. EDS of CONP- combustion

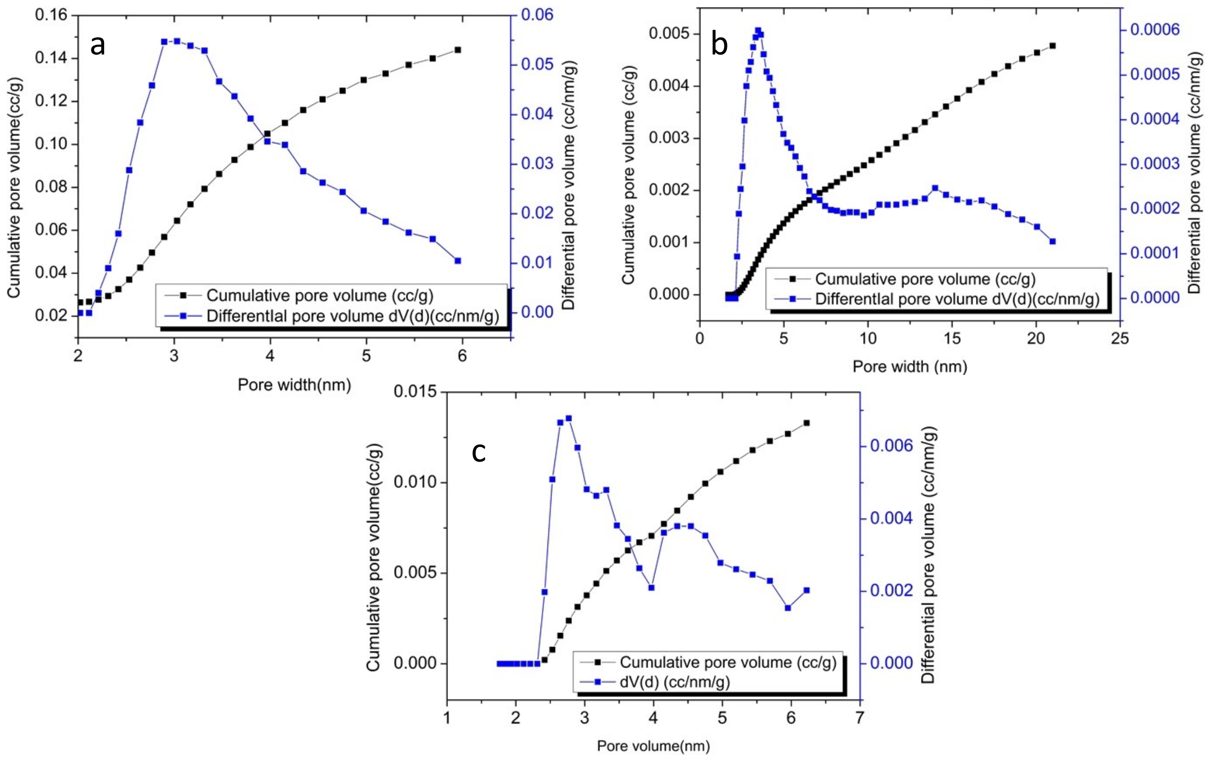

Figure S4. Pore distribution curves of (a) CONP-$NaBH_4$, (b) CONP-hydrothermal and (c) CONP-combustion

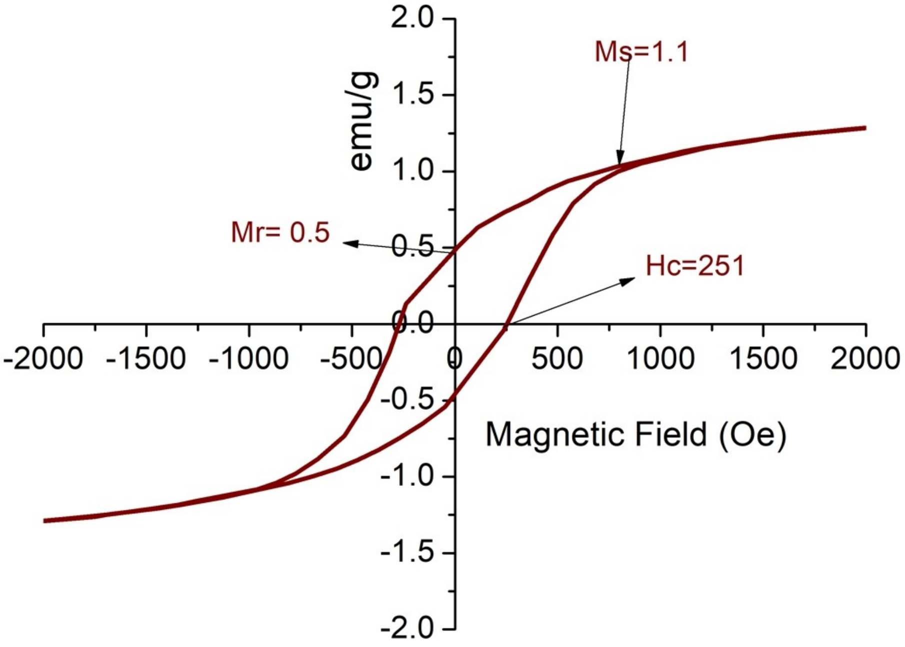

Figure S5 Magnetisation hysteresis curve for CONP-$NaBH_4$

Table S1 ICP-AES analysis CONP-$NaBH_4$

Co (%)	B (%)	Na (%)
44	4.4	1.7

Calculation S1: Calculation of CoB %

B% in CONP-$NaBH_4$ = 4.7%

B% in CoB= 11*100/70

%CoB in CONP-$NaBH_4$ =4.7 *70*100/11*100= 29.9%

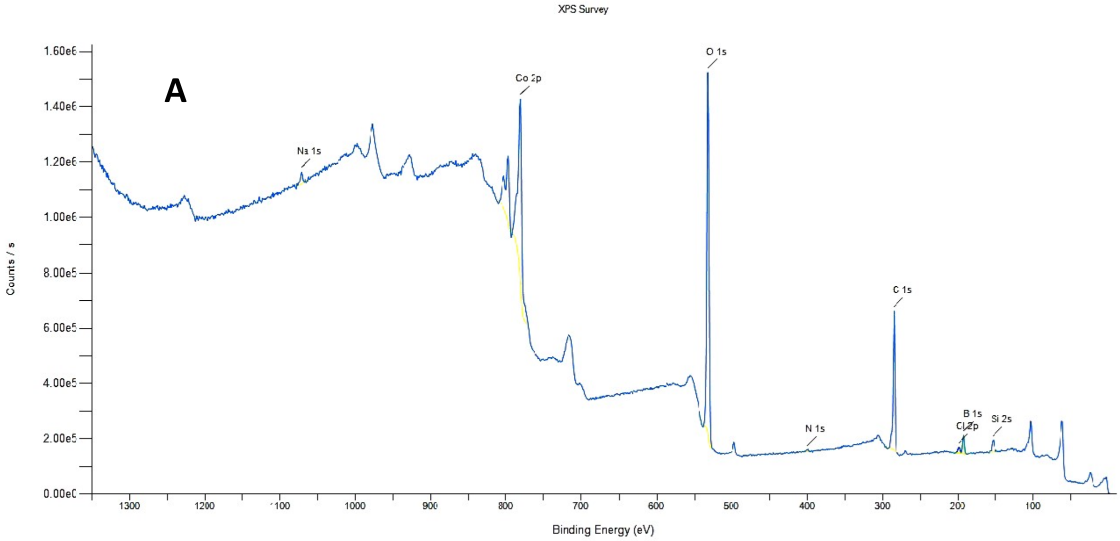

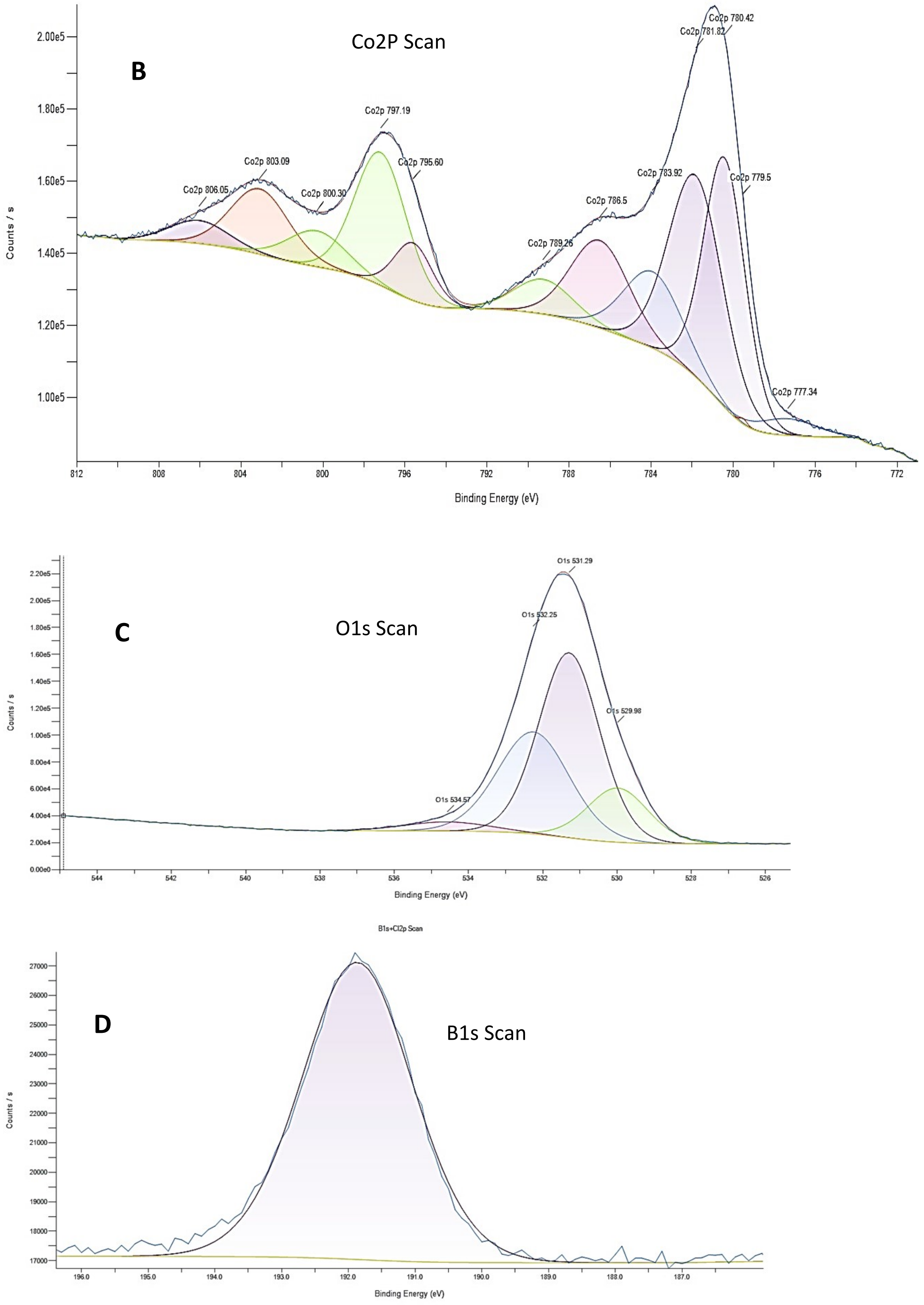

Fig. S6 XPS spectra of CONP-$NaBH_4$ (A)Survey scan, (B) Co2p scan, (C) O1s scan and (D)B1s scan

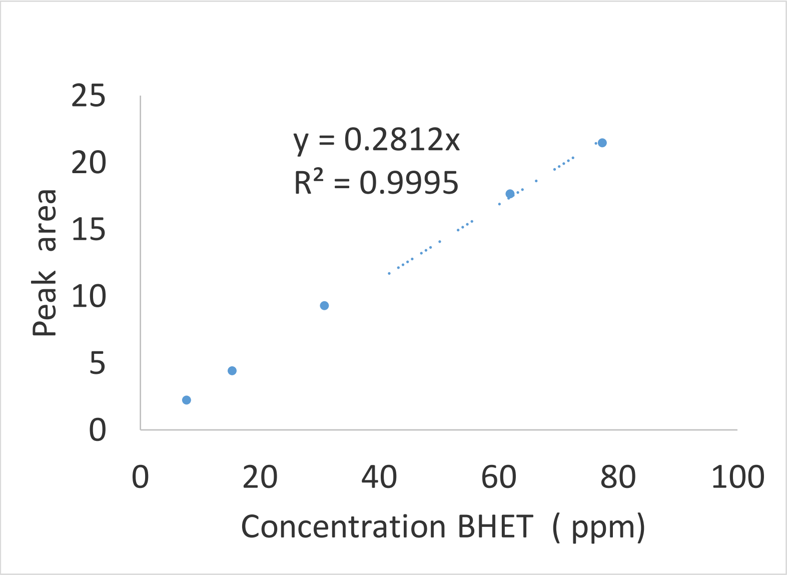

Figure S7. Calibration graph for HPLC analysis

Table S2 Mass balance summary

	Catalyst	BHET (%)	Oligomers (%)	Unreacted PET (%)	Total
1	CONP-$NaBH_4$	97	2.5	0	99.5
2	CONP-hydrothermal	70.1	5.3	24	99.1
3	CONP-combustion	82.5	3.6	14	100.4

Calculation S2: Calculation of Turn over frequency (TOF)

$$\textbf{TOF} = \frac{number\ of\ moles\ of\ \ product}{number\ of\ moles\ of\ reactant\ \ x\ time}$$

Number of moles BHET = Weight of BHET formed / Molecular weight

Weight of BHET formed from 1 g of PET in 1 hour = 1.18 g

Number of moles of BHET = 1.18/254 = 4.66 mmol

Weight of catalyst = 0.01g

Co % (surface active sites) from XPS = 10.14%

Number of moles of Co in the catalyst = (0.01 * 10.14) / (100*59) = 0.0172 mmol

TOF = 4.66 / 0.0172 **= 271 h^{-1}**

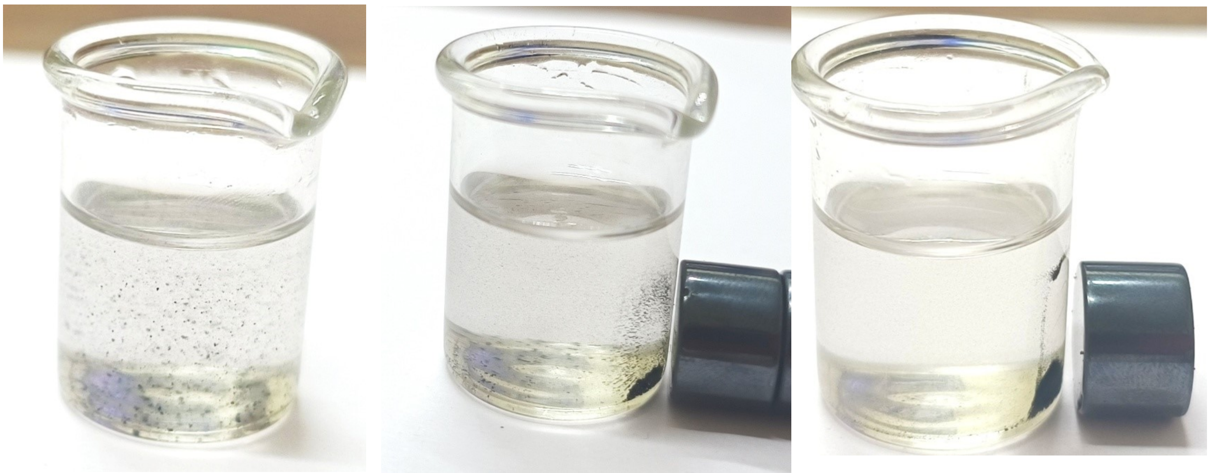

T=0 minutes T= 1 minutes T= 6 minutes

Figure S8 Separation of catalyst from 25mL EG

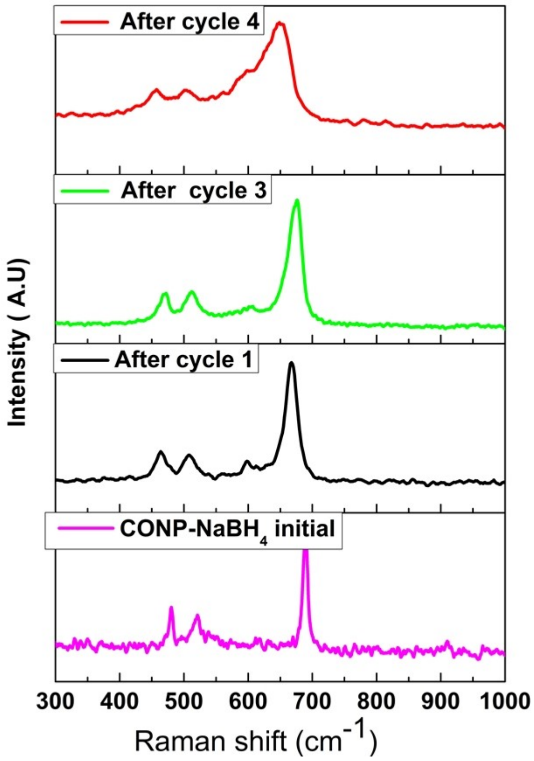

Figure S9 Raman spectra of regenerated catalyst

Table S3 Leaching out of cobalt in each regeneration cycle

Cycle number	Cobalt in the filtrate after separation of catalyst (ppm)
1	20
2	15
3	13
4	10

Hot filtration test

Hot filtration test was conducted to prove the heterogeneous catalysis. Glycolysis was carried with 0.5 g PET, 1% catalyst, and a known amount (0.083 g) of N-methyl pyrrolidone as internal standard at 180°C. After 1 hour is catalyst is filtered out while reaction mixture is hot. 20μL of sample was withdrawn and analysed for BHET % by NMR spectroscopy using DMSOd6 as the solvent. The hot filtrate is allowed to react for 1 more hour and BHET content was calculated by NMR and HPLC methods.

Calculation

A singlet at 8.00–8.15 ppm, corresponding to the aromatic protons of BHET, and a singlet at 2.7 ppm, corresponding to the methyl protons of NMP are used to calculate the BHET content.

$$BHET\ (\%) = \frac{C_{BHET\ x100}}{C_{PET}} = \frac{IBHET * 3}{INMP * 4} X \frac{Moles\ of\ NMP\ added}{moles\ of\ PET\ added}\ x\ 100$$

Here, *IBHET* and *INMP* represent the integrals of BHET at 8.1 ppm and NMP at 2.7 ppm, respectively.

Table S4 BHET % from NMR

	Reaction time (H)	NMP integral	BHET integral	BHET%
1	1	3	10.31	88.5
2	2	3	9.82	84.3

Table S5 BHET % from HPLC analysis

	Conditions	BHET (%) after 2-hour reaction
1	Hot filtration test	85%
2	Normal glycolysis (with catalyst for 2hour)	98%
